# Array-based comparative genomic hybridization for genomic-wide screening of DNA copy number alterations in aggressive bone tumors

**DOI:** 10.1186/1756-9966-31-100

**Published:** 2012-11-30

**Authors:** Masahiko Kanamori, Akimi Sano, Taketoshi Yasuda, Takeshi Hori, Kayo Suzuki

**Affiliations:** 1 Department of Human Science, University of Toyama, 2630 Sugitani, Toyama city, Toyama 930-0194, Japan; 2 Department of Orthopaedic Surgery, University of Toyama, 2630 Sugitani, Toyama city, Toyama 930-0194, Japan

**Keywords:** Osteosarcoma, Giant cell tumor, Bone tumors, Microarray, Comparative genomic hybridization

## Abstract

**Background:**

The genetic pathways of aggressive changes of bone tumors are still poorly understood. It is very important to analyze DNA copy number alterations (DCNAs), to identify the molecular events in the step of progression to the aggressive change of bone tissue.

**Methods:**

Genome-wide array-based comparative genomic hybridization (array CGH) was used to investigate DCNAs of 14 samples from 13 aggressive bone tumors, such as giant cell tumors (GCTs) and osteosarcoma (OS), etc.

**Results:**

Primary aggressive bone tumors had copy number gains of 17.8±12.7% in the genome, and losses of 17.3±11.4% in 287 target clones (threshold for each DCNA: ≦085, 1.15≦). Genetic unstable cases, which were defined by the total DCNAs aberration ≧30%, were identified in 9 of 13 patients (3 of 7 GCTs and all malignant tumors). High-level amplification of *TGFβ2*, *CCND3*, *WI-6509*, *SHGC-5557*, *TCL1A*, *CREBBP*, *HIC1*, *THRA*, *AFM217YD10*, *LAMA3*, *RUNX1* and *D22S543*, were commonly observed in aggressive bone tumors. On the other hand, *NRAS*, *D2S447*, *RAF1*, *ROBO1*, *MYB*, *MOS*, *FGFR2*, *HRAS*, *D13S319*, *D13S327*, *D18S552*, *YES1* and *DCC*, were commonly low. We compared genetic instability between a primary OS and its metastatic site in Case #13. Metastatic lesion showed increased 9 DCNAs of remarkable change (m/p ratio ≧1.3 folds), compared to a primary lesion. *D1S214, D1S1635, EXT1, AFM137XA11, 8 M16/SP6, CCND2, IGH, 282 M15/SP6, HIC1* and *LAMA3,* were overexpressed. We gave attention to *HIC1* (17p13.3), which was common high amplification in this series.

**Conclusion:**

Our results may provide several entry points for the identification of candidate genes associated with aggressive change of bone tumors. Especially, the locus 17p11-13 including *HIC1* close to *p53* was common high amplification in this series and review of the literature.

## Background

The development and progression of aggressive bone tumor is a multi-step process. The acquisition of chromosomal abnormalities in tumor cells and a series of genetic alterations occurring over the life-time of the tumor are one of the central events in malignant transformation or aggressive change. Multiple studies have identified the prevalence and clinical significance of a various genetic markers in primary bone tumors [1,2]. However, the genetic pathways of aggressive changes of bone tumors are still poorly understood. It is very important to analyze DNA copy number alterations (DCNAs), to identify the molecular events in the step of progression to the aggressive change of bone tissue.

Metaphase comparative genomic hybridization (metaphase CGH) enabled us to detect DCNAs on whole chromosomes [3,4]. But the resolution of metaphase CGH is approximately 2 Mb for amplifications and 10 − 20 Mb for deletions. Advances in mapping resolution using array-based CGH (array CGH), have greatly improved resolving power in comparison to metaphase CGH, and provide more details regarding both the complexity and exact location of genomic rearrangements leading to DCNAs [5,6]. Thereafter, array CGH technologies for identifying target molecules developed to permit for the identification of genes involved in tumors [3,4].

In this study, we investigated DCNAs of human aggressive bone tumors using the technique of array CGH. The quantitative measurement of DCNAs across the genome may facilitate oncogene identification, and might also be used for tumor classification.

## Materials and methods

### Tumor tissue specimens and DNA extraction

Fourteen bone tumor samples were collected from 13 patients with aggressive bone tumors and frozen until use. Samples from 7 giant cell tumors (GCTs), 5 osteosarcoma (OS) and 1 chondrosarcoma, were obtained from the surgical- or biopsied specimens at the University Hospital of Toyama (Table 1). Patients consisted of 6 men and 7 women with an average age of 32.9 years old (range, 7–65 years). No cases had been received the chemotherapy before the sampling. This study protocol was approved by the Institutional Review Board for Human Use at the University Hospital of Toyama.

**Table 1:** Clinicopathologic data on the samples in genomic array analysis

Cases	Age	Gender*	Diagnosis**	Follow-up***	Recurrence****	Outcome*****
1	16	F	GCT	9y	none	NED
2	16	F	GCT	12.5y	1	NED
3	18	M	GCT	11.2y	1	NED
4	21	M	GCT	11y	none	NED
5	25	M	GCT	12.3y	none	NED
6	41	F	GCT	20.6y	2	AWD
7	55	M	GCT	16.2y	2	AWD
8	47	F	chondrosarcoma	20y	none	NED
9	7	F	OS	4y	metastasis (+)	DOD
10	41	M	OS	9 m	metastasis (+)	DOD
11	58	F	OS	20y	none	NED
12	65	F	OS	6 m	metastasis (+)	DOD
13a	18	M	OS (primary)	4 m	metastasis (+)	DOD
13b			OS (metastasis)			

*Gender; F: female, M: male.

**Diagnosis; GCT: giant cell tumor, OS: osteosarcoma.

***Follow-up; m: month, y: year.

****Recurrence: The number means operation times due to the recurrences.

*****NED: no evidence of disease, AWD: alive with disease, DOD: dead of disease.

Tumor specimens were stored frozen at −80°C until use. Genomic DNA was isolated from the tumor according to standard procedures using proteinase K digestion and phenol-chloroform extraction [7].

### Hybridization and analysis of array CGH

Hybridization and analysis of array CGH were performed according to the manufacture’s protocols (Vysis-Abbott Japan Inc., Tokyo, JAPAN). The array CGH consisted of 287 clones containing important tumor suppressor and oncogene loci. Each tumor DNA sample was labeled and hybridized to microarrays for CGH. One hundred nanogram of tumor DNA was labeled by random priming with fluorolink cy3-dUTP (Perkin-Elmer Life Sciences, Inc., Boston, MA, USA), and normal reference DNA was labeled in the same fashion with cy5-dUTP. Then, the tumor and control DNAs were mixed with Cot-1 DNA (Vysis-Abbott Japan Inc), precipitated, and re-suspended in microarray hybridization buffer containing 50% formamide. The hybridization solution was heated to 80°C for 10 min to denature the DNA, and then was incubated for 1 h at 37°C. Hybridization was performed for 72 h in a moist chamber, followed by post-hybridization wash in 50% formamide/2xSSC at 45°C. Slides were mounted in phosphate buffer containing 4', 6-diamidino- 2-phenylindole (array DAPI solution). Fluorescence intensity images were obtained from the hybridized microarray slides using GenoSensor Reader System equipped with Array 300 Software (Vysis-Abbott Japan Inc.) according to the manufacture’s instructions. The total intensity and the intensity ratio of the two dyes for each spot were automatically calculated [7,8].

### Evaluation of array CGH

The diagnostic cut-off level representing gains and losses of DCNAs was set to 1.15 (upper threshold) and 0.85 (lower threshold), respectively [7,8]. The *p* value is the probability that the data value for an individual set of target spots is part of the normal distribution. All ratios were filtered by *p* values, and only those samples with *p* values of 0.01 or less were displayed in the GenoSensor Reader System.

We defined the three grades by the genomic imbalances from the data of array CGH; genetically stable group (genetic aberration <5%), intermediate group (5%≦genetic aberration <30%), genetically unstable group (genetic aberration ≧30%).

### Statistical analysis

The results are expressed as the mean ± SD. We used independent sample *t*-test for continuous variables and chi square test for categorical variables in comparison. A *p* value less than 0.05 was considered significant. All statistics were calculated using StatMate III software (Atoms Co., Tokyo, Japan).

## Results

### Overall array CGH results in aggressive bone tumors

**Figure 1:**
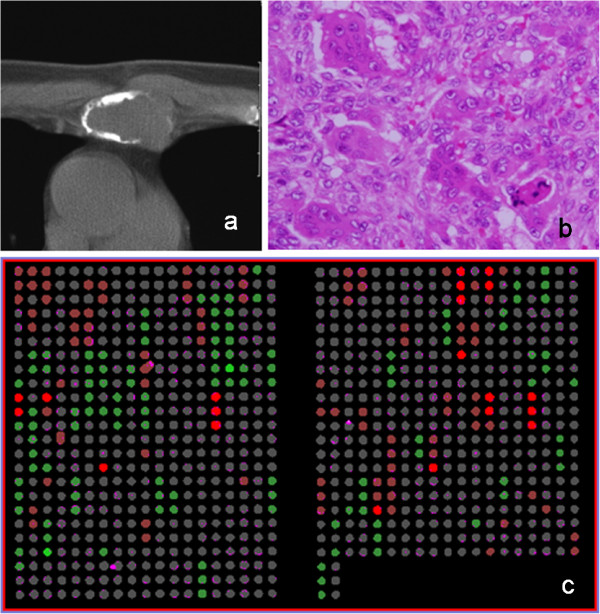
**A representative case and an array CGH slide (Case #7). a**: Radiographs of GCT originated from sternum. **b**: Histological appearance showing GCT (H&E x200). **c**: A study of microarray CGH.

**Figure 2:**
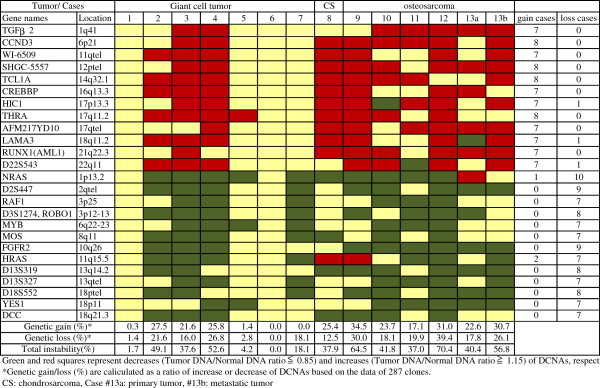
Summary of DCNAs data detected by array CGH.

Figure 1 shows a representative case, and a microarray slide which was hybridized by array CGH technique. DCNAs of primary tumors showed 17.8±12.7% in gains, and 17.3±11.4% in losses of target 287 clones. The average of the proportion of total genetic instability reached the 38.6±22.8%. Genetic unstable cases which were defined by the total DCNAs aberration (≧30%) were identified in 9 of 13 patients (3 of 7 GCTs and all malignant tumors). All malignant cases were genetically classified into the unstable group. We picked up major gene names, which showed many gain cases or loss cases. An overall array CGH results and gene names of common genetic instability are listed in Figure 2.

High-level amplification of *TGFβ2* (1q41), *CCND3* (6p21), *WI-6509* (11qtel), *SHGC-5557* (12ptel), *TCL1A* (14q32.1), *CREBBP* (16q13.3), *HIC1* (17p13.3), *THRA* (17q11.2), *AFM217YD10* (17qtel), *LAMA3* (18q11.2), *RUNX1* (21q22.3) and *D22S543* (22q11), was commonly observed in aggressive bone tumors. On the other hand, *NRAS* (1p13.2), *D2S447* (2qtel), *ROBO1* (3p12-13), *RAF1* (3p25), *MYB* (6q22-23), *MOS* (8q11), *FGFR2* (10q26), *HRAS* (11q11.5), *D13S319* (13q14.2), *D13S327* (13qtel), *YES1* (18p11), *D18S552* (18ptel) and *DCC* (18q21.3) were commonly low (Figure 2).

### Clinical relevance in GCT

**Figure 3:**
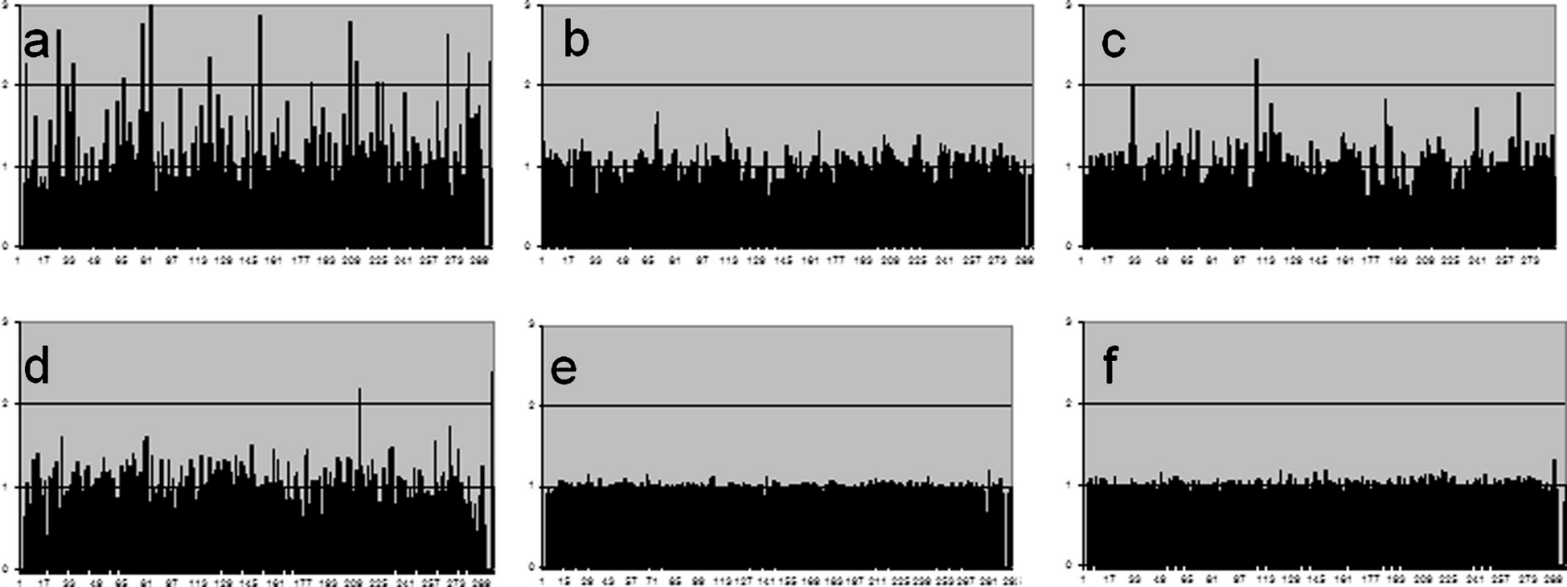
**Representative genetic unstable group (a-d) and stable group (e, f) in a study of microarray CGH. a**: Case #9 (OS), **b**: Case #10 (OS), **c**: Case #12 (OS), **d**: Case 4 (GCT), **e**: Case #2 (GCT), **f**: Case #5 (GCT).

GCT is an aggressive bone tumor, but not malignant. Seven GCT series were divided into three groups: 3 cases were genetically unstable group, and 3 cases were stable group (Figure 3). One case (Case #7) belongs to the intermediate group. Histologically, however, we could not find the difference in each GCT case. The mean clinical follow-up time of these GCT cases was 11.8 years. Tumor recurrence was observed in all cases of genetically unstable group. On the other hand, the recurrence rate of stable group was low (33.3%). However, there was no significance between two groups (chi-square test; *p* = 0.083), because the sample size was small.

As many GCTs have some telomeric associations, we have given an attention to these areas. In analyzed 73 clones of telomeric area, losses of *D2S447* (2qtel), and gain of *WI-6509* (11qtel) and *D19S238E* (19qtel) were mainly observed.

### Primary vs. Metastatic OS

**Figure 4:**
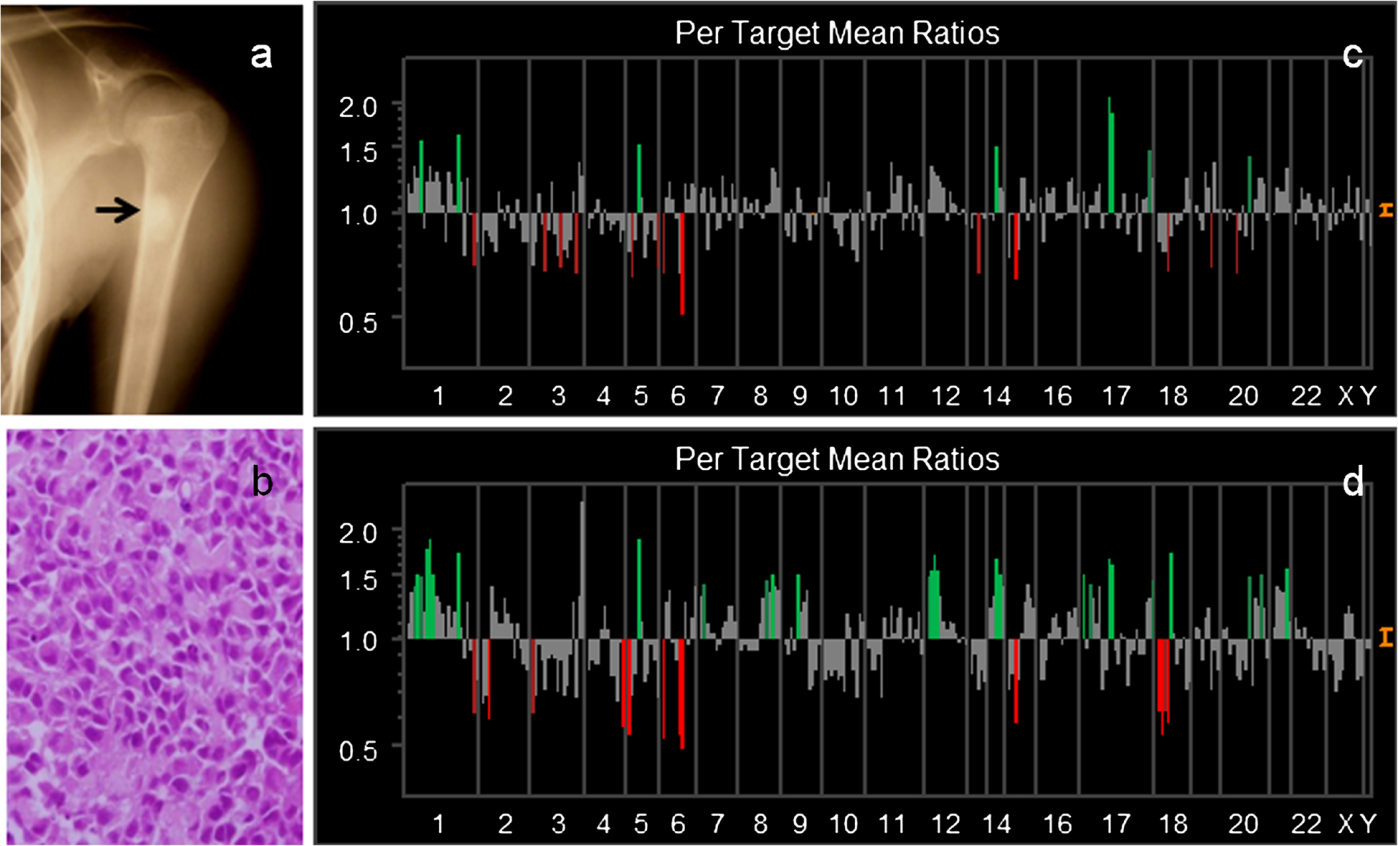
**Genetic instability analyzed by array CGH in Case #13. **Primary bone tumors showed the genetic instability of 26 DCNAs of 287 clones (**c**), whereas a metastatic lymph node showed 57 DCNAs in 287 clones (**d**). The genetic aberration of metastatic lymph node is relatively high compared with a primary bone tumor. **a**: A radiogram of humerus showing the osteosclerotic change by the osteosarcoma. **b**: Histological appearance showing atypical cells with osteoid formation. **c**: A study of microarray CGH (primary tumor). **d**: A study of microarray CGH (metastatic tumor).

We compared the genetic instability of both primary OS and a metastatic lymph node in Case #13. Briefly, 18-year-old man presented with the left shoulder mass. Radiographs revealed an osteosclerotic lesion of the proximal humerus (Figure 4a). A chest radiogram and CT scans showed multiple lung metastases. A small nodule was palpable in the axillary region. We biopsied bone tumor and removed a local swelling lymph node. Histologic examination of the both samples showed osteoblastic OS (Figure 4b). Chromosomal analysis by G-band showed 77–82 chromosomes with various complicated translocation from the primary tumor.

In this case, array CGH resulted in 22.6% gain of DCNAs and 17.8% loss of primary tumor (genetic total instability; 40.4%). Chromosomal instabilities of primary tumor detected by array CGH, are figured out (Figure 4c). However, a metastatic lymph node showed the gain of 30.7%, and the loss of 26.1% of DCNAs (genetic total instability; 56.8%). Genetic aberrations of a metastatic lesion were clearly increased (Figure 4d). We picked up detected DCNAs presenting with remarkable significant gains (≧1.30) or losses (≦0.85) in a metastatic sample compared to a primary sample (m/p ratio), and listed in Table 2. Thirty-one DCNAs of 287 clones were gained. Of these, 12 DCNAs also showed high level amplification in the primary site.

**Table 2:** Genetic instability between primary and metastatic tumor at Case #13

	**location**	**Gene name**	**metastasis (≧1.30)**	**primary tumor**	**m/p ratio**
**1p**	1p36	CDC2L1(p58)	1.39	1.33	1.05
	1p36.33	PPKCZ	1.52	1.24	1.23
	1p36.33	TP73	1.48	1.58	0.94
	1p36.31	D1S214	1.76	1.21	1.45*
	1p36.22	D1S1635	1.88	1.33	1.41*
	1p36.13	D1S199	1.51	1.22	1.24
**1q**	1q21	WI-5663	1.73	1.64	1.05
**5p**	5p13	DAB2	1.87	1.55	1.21
**8q**	8q24.11-q24	EXT1	1.44	1.03	1.40*
	8q24-qter	PTK2	1.51	1.31	1.15
	8q tel	SHGC-3110	1.40	1.29	1.09
	8q tel	U11829	1.35	1.16	1.16
**9p**	9p11.2	AFM137XA11	1.52	1.16	1.31*
**12p**	12p tel	8 M16/SP6	1.49	1.08	1.38*
	12p tel	SHGC-5557	1.52	1.34	1.13
	12p13	CCND2	1.71	1.29	1.33*
	12p13.1-p12	CDLN1B(p27)	1.53	1.25	1.22
**14q**	14q32.32	AKT1	1.68	1.51	1.11
	14q tel	IGH(D14S308)	1.51	1.16	1.30*
	14q tel	IGH(SHGC-36156)	1.39	1.14	1.22
**17p**	17p tel	282 M15/SP6	1.52	1.14	1.33*
	17p13.3	HIC1	1.42	1.04	1.37*
	17p13.1	TP53(p53)	1.40	1.19	1.18
	17p12-17p11.2	LLGL1	1.67	2.06	0.81*
	17p12-17p11.2	FLI, TOP3A	1.60	1.88	0.85*
**18q**	18q11.2	LAMA3	1.73	0.87	1.99*
**20q**	20q13.1-q13.2	PTPN1	1.46	1.43	1.02
	20q13	TNFRSF6B(DCR3)	1.50	1.23	1.22
**21q**	21q22.3	RUNX1(AML1)	1.40	1.16	1.21
	21q22	DYRK1A	1.37	1.13	1.21
	21q tel	PCNT2(KEN)	1.56	1.30	1.20

*m/p ratio: the ratio of DCNAs between the primary (p) and metastatic (m) tumor (≧1.30 or ≦0.85). No DCNAs of the loss (≦0.85) was detected in the metastasis.

It is important to assess the change of DCNAs between a metastatic tumor and a primary tumor. Nine DCNAs (m/p ratio ≧1.30 folds) showed remarkable enhancement, compared to a primary lesion; *D1S1635* (1p36.22), *D1S214* (1p36.31), *EXT1* (8q24.11-q24), *AFM137XA11* (9p11.2), *CCND2* (12p13), *8M16SP6* (12ptel), *IGH* (14qtel), *HIC1* (17p13.3) and *LAMA3* (18q11.2), *282 M15/SP6* (17ptel). On the other hand, loss of DCNAs (≦0.85) in a metastatic sample, was only *LLGL1* (m/p ratio = 0.81) and *FLI (TOP3A)* (m/p ratio = 0.85). Both of these genes are encoded on the location of 17p11.2-17p12. These DCNAs showing remarkable enhancement or decreasing, may provide several entry points for the identification of candidate genes associated with metastatic ability.

## Discussion

Our present analysis indicated to 25 genes showing genetic instability, as target genes of aggressive bone tumors (Figure 2). Especially, the loss of *NRAS* was mainly observed in 10 cases (76.9%) of 13. *NRAS* mutations have detected prostate cancers before [9]. However, there has been no report about the relationship between bone tumors and *NRAS*.

The incidence of aggressive changes of bone tissue is low. Similar to other solid tumors, malignant changes are characterized by high propensity for metastasis. Metaphase CGH studies have identified frequent gains and amplifications at 1p21-32, 1q21-24, 5p13, 6p12, 8q23-24, 8cen-q13, 17p11.2-13, 19q, and Xp21, and frequent losses at 6q16, 10p12-pter, and 10q22-q26 in OS [2,10-13]. Recent studies have also reported that amplification at 17p11.2-ptel has been found in approximately 13-29% of high-grade OS [11,14,15].

In our data, the most remarkable change in metastatic tumor was occurred at increases (≧1.30) of *D1S1635* (1p36.22), *D1S214* (1p36.31), *EXT1* (8q24.11-q24), *AFM137XA11* (9p11.2), *CCND2* (12p13), *8 M16/SP6* (12ptel), *IGH* (D14S308), *HIC1* (17p13.3), *282 M15/SP16* (17ptel), and *LAMA3* (18q11.2). DCNAs of *p53* (17p13.1) have also increased scarcely (1.19 → 1.40), which have been suggested as an OS-related gene. As Chen, et al. [16] suggested, *HIC1* (hypermethylated in cancer-1 located at 17p13.3) was frequent with *p53* mutations in human OS. Their results indicated the importance of genes altered only through epigenetic mechanisms in cancer progression in conjunction with genetically modified tumor suppressor genes. In our study, *HIC1* was also higher in the metastatic lesion than the primary site (m/p ratio =1.37 in Table 2). Therefore, we gave attention to the locus of 17p13 including *HIC1* as a target gene.

Recent studies have reported that overexpression of 17p11-p12 have been linked p53 degradation [10,16-20]. In Case #13, the gain of *LLGL1, FLI (TOP3A)* at 17p11-p12 have also detected. However, these two DCNAs were decreased in a metastatic sample, compared with primary tumor, which might be important in the step of metastasis. These findings support that target genes close to *p53* (17p13.1), may contribute to OS tumorigenesis [17,18].

Thus, the present pilot study suggests that array CGH could powerful means to detect genetic instability and gene aberrations that are reflected to the progression and outcome of primary aggressive bone tumors. *HIC1* is increased at the both step of aggressive change and metastatic process. *HIC1* might play a role of bone tumor progression and metastasis. We should pay attention the locus of 17p11-13 including *HIC1, LLGL1, FLI (TOP3A)*, as well as *p53*. Further detailed studies are necessary to clarify genetic pathways of the aggressive bone tumors.

## Conclusion

Our results may provide several entry points for the identification of candidate genes associated with aggressive change of bone tumors. Especially, the locus 17p11-13 including *HIC1* close to *p53* was common high amplification in this series and review of the literature.

## Competing interests

The authors declare that they have no competing interests.

## Authors’ contributions

MK participated in the data collection, performed the statistical analysis and drafted the manuscript.

AS, TY and TH made substantial contributions to the analysis and interpretation of data. KS helped to draft the manuscript. All authors read and approved the final manuscript.
